# Neutrophil-to-lymphocyte ratio and platelet-to-lymphocyte ratio as predictive markers in hepatoblastoma

**DOI:** 10.3389/fped.2023.904730

**Published:** 2023-04-12

**Authors:** Tan Xie, Dongliang Hou, Jiaxiang Wang, Song Zhao

**Affiliations:** ^1^ Pediatric Surgery Department, The First Affiliated Hospital of Zhengzhou University, Zhengzhou, China; ^2^ Pediatric Surgery, Henan Children's Hospital, Zhengzhou, China; ^3^ Thoracic Surgery Department, The First Affiliated Hospital of Zhengzhou University, Zhengzhou, China

**Keywords:** hepatoblastoma, neutrophil-to-lymphocyte ratio, platelet-to-lymphocyte ratio, prognostic, markers

## Abstract

**Background:**

The neutrophil-to-lymphocyte ratio (NLR) and platelet-to-lymphocyte ratio (PLR) have been presented to be a prognostic indicator in several cancers. We were supposed to evaluate the prognostic role of such inflammatory markers in hepatoblastoma (HB).

**Methods:**

Total of 101 children, diagnosed with hepatoblastoma between January 2010 and January 2018, were enrolled before treatment in the study. The clinicopathological parameters, and outcomes were collected through laboratory analyses and patient follow-up. The association between NLR, PLR, and clinicopathological characters were analyzed with Wilcoxon test, Chi-Squared test, Kaplan-Meier, Log-rank and Cox regression analyses.

**Results:**

NLR and PLR were significantly elevated in HB patients (*P* < 0.001), and related to age (*P* < 0.001), risk stratification system (*P* < 0.001), and pretreatment extent of disease (*P *< 0.0001). NLR was significantly related to alpha-fetoprotein (*P* = 0.034) and lactate dehydrogenase (*P* = 0.026). The 3-year overall survival (OS) and event-free survival (EFS) were poor in the high-NLR group (OS: 44.3% vs. 90.3%, *P *< 0.0001, EFS: 38.6% vs. 80.6%, *P* = 0.0001). The 3-year OS and EFS were poor in the high-PLR group (OS: 49.1% vs. 68.8%, *P* = 0.016, EFS: 39.6% vs. 64.6%, *P* = 0.0117). The multivariate analysis suggested that NLR (HR: 11.359, 95% CI: 1.218–105.947; *P* = 0.033) and risk stratification (HR: 44.905, 95% CI: 2.458–820.36; *P* = 0.01), were independent predictors of OS.

**Conclusion:**

Our research showed that elevated NLR and PLR were the poor prognostic factors in HB patients before treatment. The NLR was an independent prognostic factor for OS.

## Introduction

Primary hepatic malignancies among children are relatively rare, accounting for approximately 1% of new cancer diagnosis in childhood. Hepatoblastoma (HB) occurs at an incidence of 0.5–1.5 per 100,000 population, which accounts for about 80% of the malignant liver tumors in children, younger than 3 years (1). Treatment of hepatoblastoma includes chemotherapy, radiotherapy, surgical resection, immunotherapy and liver transplantation (2). The survival outcomes have been improved significantly during the past four decades. Effective HB treatment requires complete tumor resection. According to the International Childhood Liver Tumors Strategy Group (SIOPEL), about 90% of patients can be treated with radical surgery and the 5-year overall survival rate for HB can reach 75%. Liver transplantation is the only way to prolong survival in the remaining 10% of children with unresectable tumors (3, 4). In the previous reports, tumor stage, alpha-fetoprotein (AFP), histological type, type of radical surgery, metastasis, and adjuvant therapy such as chemoradiation were proposed to be the prognostic indicators in hepatoblastoma (4). Because of limited patient numbers, potential risk factors were considered not to be statistically significant, the most accurate prediction of disease staging usually occurred after resection and thus there was not a better biomarker to detect HB at an early tumor stage. The application of new biomarkers to the established prognostication systems might be beneficial to the early assessment and optimal treatment of disease.

Some literature reports that the systemic inflammatory response is involved in the process of tumor occurrence, invasion and metastasis. The systemic inflammatory response is manifested by the increase of neutrophils, platelets and relative lymphopenia detected in Peripheral blood count examination (5). Activated neutrophils and platelets promote tumor cell proliferation and metastasis by activating angiogenic, epithelial, and stromal growth factors in the tumor microenvironment (5, 6). In addition, unbalanced immune response and lymphocytopenia might reflect an impaired T lymphocyte-mediated antitumor response (7). Therefore, comprehensive analysis of neutrophils, platelets, and lymphocytes can better determine the progression and prognosis of malignant tumors (8). Moreover, these indicators are easily available and affordable through routine laboratory tests. Neutrophil–lymphocyte ratio (NLR) and platelet-to-lymphocyte ratio (PLR) are calculated as neutrophil and platelet counts divided by lymphocyte counts. Those combined calculations are considered to be comparatively more stable than the absolute counts, and they could reflect the changes in inflammatory factors and immune cells (9).

Several studies demonstrated that elevated ratio of peripheral NLR and PLR, in patients with different types of cancers, were associated with a poor prognosis (10–12). However, there are little data in literature regarding the prognostic significance of the NLR and PLR for HB patients. Therefore, we conducted this research to investigate the associations between pretreatment NLR/PLR and other clinical parameters, meanwhile, investigated whether they affect the event-free survival (EFS) and overall survival (OS) of patients with HB after hepatectomy and chemotherapy.

## Patients and methods

Between January 2010 and January 2018, the clinical data of 101 HB patients who were diagnosed by histology, cytology, and typical imaging findings in the First Affiliated Hospital and Children's Hospital of Zhengzhou University were retrospectively collected. All of the patients were treated in accordance with the guidelines from SIOPEL and Children's Oncology Group (COG) risk stratification system for children with hepatoblastoma. Very low risk category [ie, well-differentiated fetal histology with pretreatment extent of disease (PRETEXT) I/II], recommends upfront resection with no chemotherapy. Low-risk patients receive upfront resection and then adjuvant chemotherapy. Intermediate-risk and high-risk individuals receive neoadjuvant therapy followed by resection and adjuvant chemotherapy (13). Patients who underwent surgical excision and chemotherapy were selected for the study. Patients age below 6 days or above 6 years with a history of active infection or chronic inflammation, those who were lost to follow-up after surgery, and premature infant were excluded. In the same period, 101 children with indirect inguinal hernia in our hospital were selected as the control group. The study was approved by the Ethics Committee of Research and Clinical Experiments of the First Affiliated Hospital of Zhengzhou University (No. KY-2021-0213).

### Blood sample and clinical variables analysis

Blood samples obtained after diagnosis of hepatoblastoma and before initial treatment were used to measure neutrophil, lymphocyte and platelet counts. Other variables, including AFP, lactate dehydrogenase (LDH), aspartate transaminase (AST) and alanine aminotransferase (ALT) were simultaneously obtained. Blood NLR and PLR were calculated as the ratio of the total count of neutrophils and platelets divided by the total count of lymphocytes.

The following clinical variables were included for analysis: age, gender, risk stratification system, AFP, AST, ALT, histologic type. HB patients are not routinely staged by the standard TNM staging system, however other systems such as the PRETEXT and COG staging schemes are used (14).

### Statistical analysis

Statistical analyses were performed using SPSS version 17.0 and GraphPad Prism version 5.0. The Wilcoxon test was used to compare two sets of quantitative data. The comparision of categorical variables between two groups was performed using the chi-squared test. The Kaplan–Meier method was used to calculate survival curves. Survival outcomes between groups were compared with the log-rank test. Variables that proved to be significant in the univariate analysis were used for performance of the multivariate Cox regression analysis. The *P*-value < 0.05 was considered to be significant. EFS was defined as the period from the start with the first treatment after the diagnosis of hepatoblastoma to disease progression, death, or the occurrence of a second malignant tumor. OS was defined as the period from the start with the first treatment after the diagnosis of hepatoblastoma to death or death regardless of the cause.

## Results

### Optimum cutoffs of the PLR and NLR

**Figure 1:**
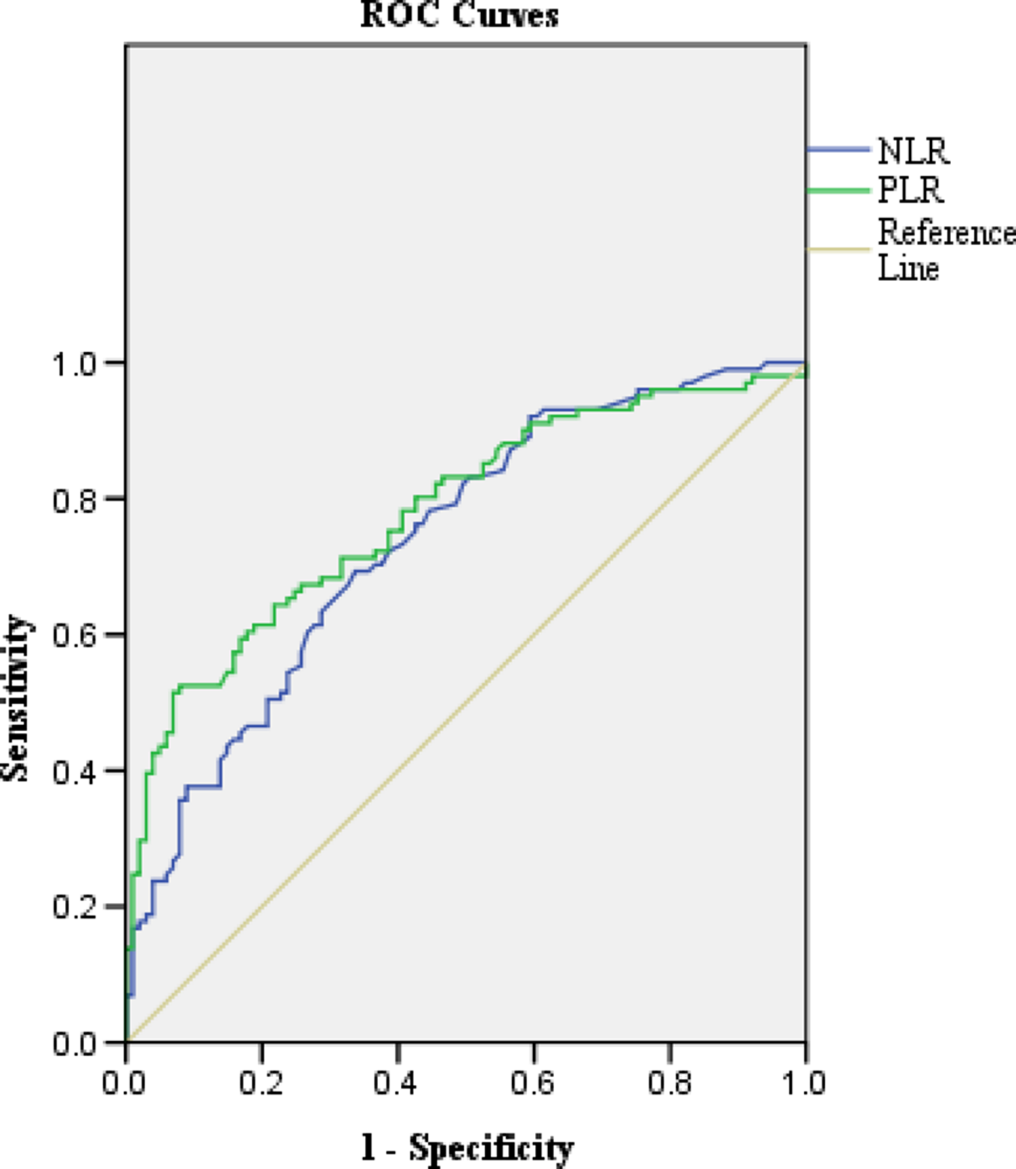
ROC analysis of NLR and PLR, as predictive values in hepatoblastoma.

The optimal cut-off values for NLR and PLR were estimated by receiver operative curve (ROC) analysis (15). Based on the AUROC curve, the NLR and PLR cut-off points were defined as 0.59 and 106.94 (AUROC: 0.739 and 0.78, respectively, Figure 1). NLR ≥ 0.59 and PLR ≥ 106.94 were considered to be elevated (High-NLR and High-PLR), while NLR < 0.59 and PLR < 106.94 were reduced (Low-NLR and Low-PLR).

### Comparisons of NLR and PLR between case group and control group

The level of NLR before treatment was higher in case group than those in the control group. There were significant differences between two groups (*P* < 0.001, Table 1). The PLR in the case group was significantly higher than that in the control group before treatment (*P* < 0.001, Table 2).

**Table 1:** Comparison between case and control groups of NLR with Wilcoxon test.

Group	*M* (P_25_, P_75_)	*Z* value	*P* value
Case group	0.91 (0.525, 1.475)	5.874	<0.001*
Control group	0.47 (0.32, 0.77)		

NLR, neutrophil-to-lymphocyte ratio.

* Statistically significant (*P *< 0.05).

**Table 2:** Comparison between case and control groups of NLR with Wilcoxon test.

Group	*M* (P_25_, P_75_)	*Z* value	*P* value
Case group	107.84 (69.5, 154.42)	6.884	<0.001*
Control group	62.5 (47.675, 80.59)		

PLR, platelet-to-lymphocyte ratio.

* Statistically significant (*P *< 0.05).

### Clinicopathologic characteristics

This study was conducted in a retrospective cohort of 101 patients with HB. The clinical characteristics of patients classified as high/low NLR and PLR are presented in Table 3. The median age of the patients in this series was 12 (range 0.3–63) months. 60 (59.41%) patients were male, and the pathological type in 62.38% of patients was epithelioid. The PRETEXT stage I, II, III and IV patients were accounted for 10.89%, 29.70%, 39.60% and 19.80%, respectively. There were 73 (72.28%) patients with low and extremely high levels of AFP (<100 or >10,000 ng/ml). More than half (64.36%) of the patients had a LDH > 300 U/L, while the patients with AST and ALT > 40 U/L were accounted for 82.18% and 13.86%, respectively.

**Table 3:** Association of the patients’ characteristics with the platelet-to-lymphocyte and neutrophil-to-lymphocyte ratios.

Characteristics		All	Low NLR	High NLR	*X*^2^ value	*P* value	Low PLR	High PLR	*X*^2^ value	*P* value
		*n* (%)	*n* (%)	*n* (%)			*n* = 48 (%)	*n* = 53 (%)		
Age	≤12 months	62 (61.39)	28 (45.16)	34 (54.84)	14.088	<0.001*	39 (62.90)	23 (37.10)	15.227	<0.001*
	>12 months	39 (38.61)	3 (7.69)	36 (92.31)			9 (23.08)	30 (76.92)		
Gender	Male	60 (59.41)	21 (35)	39 (65)	1.289	0.256	27 (45)	33 (55)	0.378	0.539
	Female	41 (40.59)	10 (24.39)	31 (75.61)			21 (51.22)	20 (48.78)		
Risk stratification	Standard risk group	38 (37.62)	24 (63.16)	14 (36.84)	30.184	<0.001*	30 (78.95)	8 (21.05)	24.12	<0.001*
	High risk group	63 (62.38)	7 (11.11)	56 (88.89)			18 (28.57)	45 (71.43)		
PRETXET stage	I	11 (10.89)	8 (72.73)	3 (27.27)	35.978	<0.001*	9 (81.82)	2 (18.18)	31.284	<0.001*
	II	30 (29.70)	18 (60)	12 (40)			24 (80)	6 (20)		
	III	40 (39.60)	2 (5)	38 (95)			8 (20)	32 (80)		
	IV	20 (19.80)	3 (15)	17 (85)			7 (35)	13 (65)		
AFP (ng/ml)	100–10,000	28 (27.72)	13 (46.43)	15 (53.57)	4.509	0.034*	17 (60.71)	11 (39.29)	2.702	0.1
	<100 or >10,000	73 (72.28)	18 (24.66)	55 (75.34)			31 (42.47)	42 (57.53)		
LDH (U/L)	≤300	36 (35.64)	16 (44.44)	20 (55.56)	4.973	0.026*	20 (55.56)	16 (44.44)	1.447	0.229
	>300	65 (64.36)	15 (23.08)	50 (76.92)			28 (43.08)	37 (56.92)		
AST (U/L)	≤40	18 (17.82)	4 (22.22)	14 (77.78)	0.334	0.563	7 (38.89)	11 (61.11)	0.655	0.418
	>40	83 (82.18)	27 (32.53)	56 (67.47)			41 (49.40)	42 (50.60)		
ALT (U/L)	≤40	87 (86.14)	28 (32.18)	59 (67.82)	0.248	0.619	41 (47.13)	46 (52.87)	0.04	0.842
	>40	14 (13.86)	3 (21.43)	11 (78.57)			7 (50)	7 (50)		
Pathological type	Epithelial type	63 (62.38)	23 (36.51)	40 (63.49)	2.662	0.103	33 (52.38)	30 (47.62)	1.583	0.208
	Mixed type	38 (37.62)	8 (21.05)	30 (78.95)			15 (39.47)	23 (60.53)		

NLR, neutrophil-to-lymphocyte ratio; PLR, platelet-to-lymphocyte ratio; PRETEXT, pretreatment extent of disease; AFP, alpha-fetoprotein; LDH, lactate dehydrogenase; AST, aspartate transaminase; ALT, alanine aminotransferase.

* Statistically significant (*P *< 0.05).

### Standard- vs. high-risk tumors

Based on the risk stratification criteria of the SIOPEL, the children were divided into the standard risk group and the high-risk group. High risk HB criteria include patients with preoperative PRETEXT IV, serum AFP < 100 ng/ml, distant metastasis at diagnosis, invasion of the portal vein, inferior vena cava or hepatic vein and tumor rupture or intraperitoneal hemorrhage (4, 16). There were 38 standard risk patients with 1 death (mortality, 2.6%). At least one high-risk factor in 63 patients, and 37 died (mortality, 58.7%).

### Relationship between clinicopathologic variables and NLR and PLR

The numbers of low NLR and high NLR patients were 31 and 70, respectively. The numbers of low PLR and high PLR patients were 48 and 53, respectively (Table 3). Age, risk stratification and PRETEXT stage were significantly associated with the high NLR and PLR groups (*P *< 0.001, respectively). AFP and LDH were significantly related with the high NLR group (*P* = 0.034 and *P* = 0.026 respectively).

### Prognosis analysis of NLR and PLR

**Figure 2:**
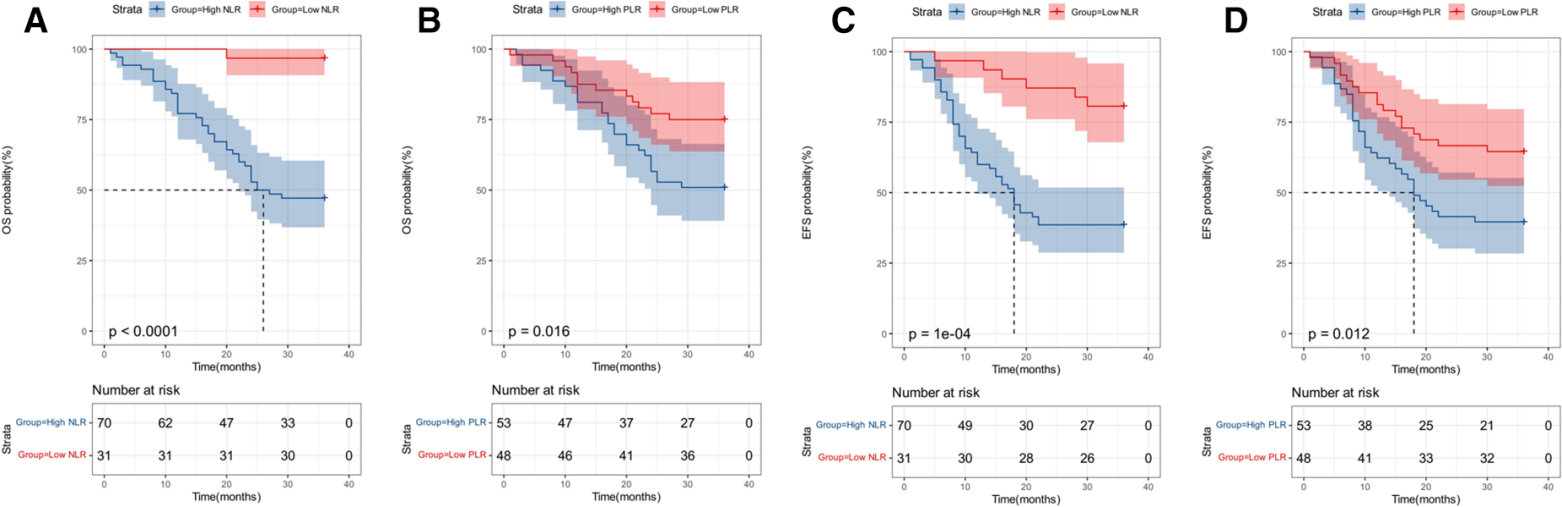
Survival analysis. (**A**) Kaplan–Meier survival curves of overall survival according to neutrophil-to-lymphocyte ratio (NLR). (**B**) Kaplan–Meier survival curves of overall survival according to platelet-to-lymphocyte ratio (PLR). (**C**) Kaplan–Meier survival curves of event-free survival according to neutrophil-to-lymphocyte ratio (NLR). (**D**) Kaplan–Meier survival curves of event-free survival according to platelet-to-lymphocyte ratio (PLR).

Patients were followed up for at least 3 years by telephone or outpatient review after discharge. The final follow-up time was January 2021. During follow-up, 49 patients (48.5%) developed tumor recurrence and 42 (41.6%) patients died due to progressive disease. For the entire study population, 3-year EFS rates was 51.5%, while 3-year OS rates was 58.4%. The 3-year OS and EFS rates of the patients with an NLR ≥ 0.59 (44.3% and 38.6%, respectively) were significantly lower than those of the patients with an NLR < 0.59 (90.3% and 80.6%, *P* < 0.0001 and *P* = 0.0001, respectively. Figures 2A,C). The 3-year OS and EFS rates of the patients with PLR ≥ 106.94 (49.1% and 39.6%, respectively) was significantly lower than those of the patients with an PLR < 106.94 (68.8% and 64.6%, *P* = 0.016 and *P* = 0.012, Figures 2B,D).

### Prognostic factors for OS

The results of the univariate analysis of different variables on 3-year OS are shown in Table 4. The 3-year OS rates of the standard risk patients (97.4%) were significantly higher than those of the high risk patients (41.3%, *P* < 0.0001). The 3-year OS rates of the patients in PRETEXT stage I and II (95.1%) were significantly higher than those of the patients in PRETEXT stage III and IV (40%, *P* < 0.0001). The 3-year OS rates of the patients with an AFP 100–10,000 ng/ml (78.6%) were significantly higher than those of the patients with an AFP < 100 or >10,000 ng/ml (56.2%, *P *= 0.0344). The 3-year OS rates of the patients with an LDH ≤ 300 U/L (80.6%) were significantly higher than those of the patients with an LDH > 300 U/L (53.8%, *P* = 0.0241).

**Table 4:** Univariate and multivariate analyses of factors for the prediction of overall survival.

Variable	Univariable analysis		Multivariable analysis	
	HR (95% CI)	*P* value	HR (95% CI)	*P* value
Age	0.5109 (0.2605–1.002)	0.0507		
Gender	0.5462 (0.2888–1.033)	0.0628		
Risk stratification	0.1616 (0.0845–0.3092)	<0.0001*	44.905 (2.458–820.360)	0.01*
PRETEXT stage	0.1615 (0.08459–0.3084)	<0.0001*	0.464 (0.047–4.564)	0.510
AFP	0.4743 (0.2376–0.9468)	0.0344*	2.203 (0.884–5.491)	0.09
LDH	0.4678 (0.2418–0.9052)	0.0241*	1.470 (0.656–3.290)	0.349
AST	0.6084 (0.2811–1.317)	0.2071		
ALT	0.7709 (0.3164–1.878)	0.5668		
Pathological type	1.411 (0.7433–2.677)	0.2926		
NLR	0.2169 (0.1114–0.4222)	0.0001*	11.359 (1.218–105.947)	0.033*
PLR	0.4539 (0.2386–0.8633)	0.016*	0.528 (0.253–1.102)	0.089

NLR, neutrophil-to-lymphocyte ratio; PLR, platelet-to-lymphocyte ratio; PRETEXT, pretreatment extent of disease; AFP, alpha-fetoprotein; LDH, lactate dehydrogenase; AST, aspartate transaminase; ALT, alanine aminotransferase; 95% CI, 95% confidence interval; HR, hazard ratio.

* Statistically significant (*P* < 0.05).

The multivariate analysis revealed that a pretherapy NLR < 0.96 [hazard ratio (HR): 11.359, 95% CI: 1.218–105.947; *P* = 0.033] was associated with longer OS than an NLR ≥ 0.96. Compared with standard risk patients, the high risk patients showed a trend towards having worse OS (HR: 44.905, 95% CI: 2.458–820.360; *P* = 0.01). The multivariate analysis confirmed that NLR, risk stratification were independent predictors of OS. AFP, LDH, PRETEXT stage, PLR were not the independent prognostic factors (Table 4).

## Discussion

The inflammation response plays an important role in the occurrence and development of tumors (5). Inflammatory cells such as neutrophils, which was attracted by chemokines and cytokines secreted from tumor cells, could infiltrate the tumor microenvironment and its interaction with tumor necrosis factor-alpha (TNF-α) and interleukins could enhance tumor immune response leading to tumor cell proliferation (17–19). Additionally, neutrophils could promote tumor progression *via* the transforming growth factor-beta (TGF-β) signaling pathway (20, 21). The lymphocyte response is an important factor in tumor immune responses, and it plays a key role in inhibiting tumor growth and progression. In the early stage of tumorigenesis, an imbalanced immune system manifests as lymphocytopenia and impaired immune response which compromises the effectiveness of host-tumor immune responses and promotes the further proliferation, diffusion and metastasis of tumor cells (22). Platelets might participate in tumor growth and metastasis through inflammatory and non-inflammatory mechanisms. Activated platelets not only regulate the function of neutrophils, lymphocytes and endothelial cells, but also promote the movement of neutrophils to inflammatory areas to induce tumor immune response (23). Platelets may also contribute to the growth and spread of malignant tumors through noninflammatory mechanisms such as promoting neo angiogenesis and attachment of tumor cells to endothelial cells, increasing microvessel permeability and extravasation of tumor cells (24, 25). In addition, the early metastatic niches formed by platelets, neutrophils and disseminated tumor cells could accelerate tumor invasion and metastasis (26). Therefore, estimated ratios such as NLR and PLR rather than absolute neutrophil, platelets and lymphocyte counts are more responsive to changes in the tumor microenvironment and tumor immune response. The high NLR and PLR indicated an imbalance immune response, which disrupted the normal anti-tumor functions and led to tumor occurrence and metastasis (27). We found that the ratio of NLR and PLR in peripheral blood of children with HB was significantly higher than that of the control group (Tables 1, 2).

NLR and PLR as an evaluation index has been widely applied in various types of solid tumors. Prabawa et al. reported that the NLR and PLR could be used as a potential biomarker for predicting the cervical cancer invasiveness (28). Xia et al. first revealed NLR was found to be an independent prognostic factor for OS and EFS in patients with osteosarcoma (29). Neofytou et al. showed that the PLR, as an adverse prognostic factor, was superior to the NLR in patients with liver-only colorectal metastases (30). In this study, we found that pretreatment NLR and PLR were significantly associated with poor OS and EFS in HB patients, which was similar to other foundings in various cancers. We also identified NLR as an independent prognostic factor for OS in multivariate analysis and we supposed that NLR might have more predictive value than PLR. To our knowledge, this is the first study to demonstrate the prognostic value of both pretreatment NLR and PLR in predicting HB.

The traditional factors: AFP, PRETEXT stage, risk stratification and the presence of metastases have long been identified as major prognostic factors for hepatoblastoma (31). The univariate analysis revealed that those factors, such as high- risk group, low or high AFP level and PRETEXT III and IV, were negative prognostic factors in patients and risk stratification was an independent prognostic factor for OS in our study. The SIOPEL identifies standard and high-risk HB with stratification to different chemotherapy groups (32, 33). In this study, we indicated that the mortality of patients with high-risk tumors was almost 20 times as high as the standard risk group, which indicating that patients with high-risk stratification would get the worse prognosis and lower survival rate. We found that 88.9% patients in the high-risk group had NLR greater than 0.59 and 71.43% patients with PLR greater than 106.94. At the same time, only 36.84% and 21.05% of low-risk patients had high NLR and high PLR. These findings suggest that NLR, PLR can be considered an indicator of risk stratification for HB. Also, we found that patients in high-risk group with higher NLR and PLR had poorer OS than standard risk patients with low NLR and PLR. This suggests that serum biomarkers, such as NLR and PLR, may be important biomarkers to further stratify patients within conventional risk stratification.

The PRETEXT system, a preoperative surgical staging system, which was based on the anatomy of the liver, was a relatively objective diagnostic method to assess the extent of the tumor (32, 33). LDH is a sensitive indicator of tumor burden and metabolic status *in vivo*, and its increase can indirectly reflect the size of tumor volume and metabolic activity in patients, often indicating that children are in advanced stage and have poor prognosis (34). Moon et al. showed that the survival of patients with HB is usually correlated with the PRETEXT classification (35). Chen et al. found that the increase of LDH has been associated with poor OS in patients with colorectal cancer (36). Zhang et al. reported that the high NLR has been associated with poorer prognosis and larger tumors in patients with gastric cancer (37). A similar finding was observed in our study showing that HB patients with high LDH and PRETEXT III/IV disease had a poor prognosis and the high NLR was significantly related with risk stratification and LDH. It is suggested that patients with high NLR might have larger tumor volume, burden and increasing metabolic status. Therefore, NLR before treatment has a certain reference value for evaluating whether to surgery or chemotherapy first for new hepatoblastoma.

AFP has been widely used to monitor the recurrence and treatment effect of hepatoblastoma (38). Patients with extreme levels of AFP should always be regarded as a high-risk group from the beginning of treatment and receive a suitable treatment for high-risk patients. The high-risk patients, which with extreme levels of AFP and a poor prognosis, should receive an intensive treatment at the beginning (4). In our study, there were significant differences in the survival rates of patients with different levels of AFP with HB. This is similar to the retrospective study showing that AFP < 100 and >1.2 × 10^6^ μg/L were an indicator of poor prognosis in HB patients (16). We found that patients with high NLR might have extreme levels of AFP. There was a significant correlation between high NLR and extreme level of AFP (AFP < 100 and >10,000 ng/ml). Similar to the function of AFP, we speculate whether NLR can be used for the detection of therapeutic efficacy of hepatoblastoma and the monitoring of tumor recurrence, which still needs further research.

This study is associated with several limitations. First, this is a retrospective study, which may have led selection bias. Second, due to the low incidence of HB, the sample size that could be used was small. Patients with a raised PLR had a significantly lower OS and EFS, confirming that elevation of the PLR indicates a more aggressive clinical phenotype, while the Cox regression showed that the PLR was not an independent prognostic factor. Age as a poor prognostic factor has been proved in various malignant tumors, such as neuroblastoma and Wilms’ tumor (39, 40). This study found that age was positively correlated with NLR and PLR, but not a prognostic factor for HB. So, we speculate that the low sensitivity value of PLR and age may have been related to the small sample size. Third, our study only included age above 6 days and below 6 years. Further research is needed on children of other ages.

## Conclusion

The pretreatment NLR and PLR are associated with survival in patients with HB. NLR was identified as an independent prognostic factor for HB patients. Measuring and monitoring the NLR value of HB patients may help in assessing the treatment response and predicting recurrence. More prospective and randomized studies are needed to confirm these findings and clarify the underlying mechanism between systemic inflammation and tumor cells.

## Data Availability

The original contributions presented in the study are included in the article/Supplementary Material, further inquiries can be directed to the corresponding author/s.
